# COVID-19 Outbreak Among a University’s Men’s and Women’s Soccer Teams — Chicago, Illinois, July–August 2020

**DOI:** 10.15585/mmwr.mm6943e5

**Published:** 2020-10-30

**Authors:** Richard A. Teran, Isaac Ghinai, Stephanie Gretsch, Tracy Cable, Stephanie R. Black, Stefan J. Green, Omar Perez, George E. Chlipala, Mark Maienschein-Cline, Kevin J. Kunstman, Susan C. Bleasdale, Marielle J. Fricchione

**Affiliations:** ^1^Epidemic Intelligence Service, CDC; ^2^Chicago Department of Public Health, Illinois; ^3^University of Illinois at Chicago.

Data on transmission of SARS-CoV-2, the virus that causes coronavirus disease 2019 (COVID-19), among college athletes are limited. In August 2020, the Chicago Department of Public Health (CDPH) was notified of a cluster of COVID-19 cases among a university’s men’s and women’s soccer teams. CDPH initiated an investigation, interviewed members of both teams, and collated laboratory data to understand transmission of SARS-CoV-2 within the teams. Numerous social gatherings with limited mask use or social distancing preceded the outbreak. Transmission resulted in 17 laboratory-confirmed COVID-19 cases across both teams (n = 45), likely from a single source introduction of SARS-CoV-2 (based on whole genome sequencing) and subsequent transmission during multiple gatherings. Colleges and universities are at risk for COVID-19 outbreaks because of shared housing and social gatherings where recommended prevention guidance is not followed. Improved strategies to promote mask use and social distancing among college-aged adults need to be implemented, as well as periodic repeat testing to identify asymptomatic infections and prevent outbreaks among groups at increased risk for infection because of frequent exposure to close contacts in congregate settings on and off campus.

## Investigation and Results

University A student athletes returned to campus* during June and were required to have two negative real-time reverse transcription–polymerase chain reaction (RT-PCR) SARS-CoV-2 tests before participating in any preseason training activities. Voluntary training sessions for both soccer teams began in July. In August, a member of the men’s soccer team reported COVID-19-related symptoms to coaching staff members (Figure). The student reported attending several social gatherings with teammates in the preceding 14 days, including a birthday party and an unsanctioned soccer match between the men’s and women’s teams. Over the next 2 days, five other soccer players reported symptoms, and both teams were instructed to isolate or quarantine.^†^ Specimens were collected from symptomatic soccer players and any other persons attending the birthday party or coed soccer match. Nine of 10 tests had positive results for SARS-CoV-2. Three days later, four more soccer players received positive test results. After the university instructed both teams to test all members, including asymptomatic persons, four additional players with SARS-CoV-2 infection were identified, for a total of 17.

**FIGURE:**
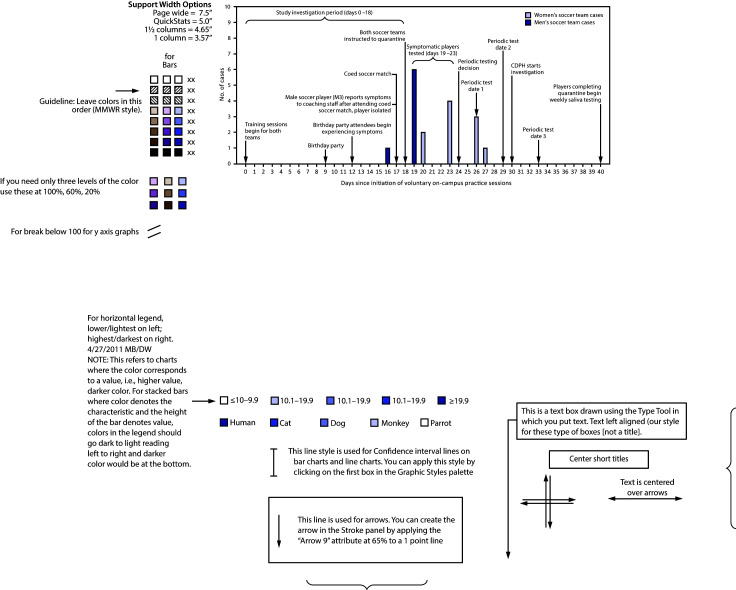
COVID-19 cases (n = 17) among a university’s* men’s and women’s soccer teams, by specimen collection date and significant events^†^ — Chicago, Illinois, July–August 2020 **Abbreviations:** CDPH = Chicago Department of Public Health; COVID-19 = coronavirus disease 2019. * On day 24, university staff members decided to begin periodic testing of all players on the men’s and women’s soccer teams to identify asymptomatic students with COVID-19 and control the outbreak. Periodic testing was performed on days 26, 29, and 33. ^^†^^ A more comprehensive timeline of events is available at https://stacks.cdc.gov/view/cdc/95822.

All specimens tested for SARS-CoV-2 within its jurisdiction are reportable to CDPH, as are all COVID-19 clusters or outbreaks in congregate settings, including universities.^§^ A case-control study was conducted to assess exposures among soccer players on both teams who participated in training sessions from the day voluntary training sessions commenced (day 0) to the day both teams were instructed to quarantine (day 18) (the investigation period).^¶^ A questionnaire was administered to collect symptom history, housing information, training details, contacts, and information on participation in social gatherings, mask use, and social distancing behaviors. Self-reported SARS-CoV-2 test results were confirmed using the university’s electronic medical record system and Illinois’ National Electronic Disease Surveillance System (I-NEDSS). A case-patient (a student with COVID-19) was defined as a person on the men’s or women’s soccer team with a positive SARS-CoV-2 RT-PCR test result who participated in training sessions during the investigation period. Controls included students on either soccer team who participated in training sessions during the investigation period and who received a negative SARS-CoV-2 RT-PCR test result during days 0–30. Logistic regression estimated odds ratios (ORs) and 95% confidence intervals (CIs) to determine the association between reported housing accommodations, social gathering attendance, and coed match participation with a positive SARS-CoV-2 test result. Analyses were performed with SAS software (version 9.4; SAS Institute). Whole-genome sequencing was conducted on available specimens to identify phylogenetic relationships based on nucleotide differences. This activity was reviewed by CDC and was conducted consistent with applicable federal law and CDC policy.**

All students who participated in training sessions during the investigation period (n = 45) were interviewed, among whom 17 SARS-CoV-2 cases were identified (attack rate = 37.8%); the other 28 students served as controls. The 45 students consisted of 21 (46.7%) players on the men’s team, 23 (51.1%) on the women’s team, and one women’s team staff member (Table). Median age was 20 years (interquartile range [IQR] = 18–21 years), 46.7% were non-Hispanic White, and 33.3% were Hispanic or Latino. Thirty-three (73.3%) students lived in shared accommodations with other teammates. In addition to the coed soccer match, 18 social gatherings were reported during the investigation period, including a birthday party, visits to friends’ dormitories or apartments, and outdoor lake gatherings. Most students (60.0%) reported attending at least one gathering. In addition, seven students who reported not attending any social gatherings were listed as event contacts by other teammates. All 17 students with COVID-19 attended at least one gathering. Among the students with COVID-19, the median number of days from the last negative SARS-CoV-2 RT-PCR test and the first positive test result was 25.0 days (IQR = 22.0–26.5 days). Eleven of the students with COVID-19 reported symptoms. None of the students were hospitalized. Compared with controls, students with COVID-19 had increased odds of living in shared accommodations with teammates (OR = 10.4; 95% CI = 1.2–89.6).

**TABLE:** Characteristics of student athletes on the men's and women's soccer team included in the COVID-19 outbreak investigation — Chicago, Illinois, July–August 2020

Characteristic	No. (column %)			Unadjusted OR (95% CI)
	All (n = 45)	Cases (n = 17)	Controls (n= 28)	
Housing status*				
Shared housing	33 (73.3)	16 (94.1)	17 (60.7)	10.4 (1.2–89.6)
Other	12 (26.7)	1 (5.9)	11 (39.3)	Reference
**Roommates with a COVID-19 patient**				
Yes	21 (46.7)	11 (64.7)	10 (35.7)	3.3 (0.9–11.6)
No	24 (53.3)	6 (35.3)	18 (64.3)	Reference
**Attendance at coed soccer match**				
Yes	28 (62.2)	12 (70.6)	16 (57.1)	1.8 (0.5–6.5)
No	17 (37.8)	5 (29.4)	12 (42.9)	Reference
**Attendance at any social gathering^†^**				
Yes	27 (60.0)	13 (76.5)	14 (50.0)	3.3 (0.8–12.5)
No	18 (40.0)	4 (23.5)	14 (50.0)	Reference

**Abbreviations:** CI = confidence interval; COVID-19 = coronavirus disease 2019; OR = odds ratio.

* Student athletes living in shared accommodations include 19 persons who lived in off-campus apartments and 14 who lived in on-campus dormitories. Student athletes with a housing status of “other” include 10 persons who lived with family and commuted to campus and two who lived in apartments or dormitories but did not have a roommate during the exposure period.

^†^ Characteristic is based on self-reported attendance at any event and does consider whether the athlete was listed as a contact by another athlete.

Probable exposure periods and elicitation windows (the time frame during which a student with COVID-19 was likely infectious and not in isolation)^††^ were determined for each student with COVID-19 based on laboratory data and symptom history (Supplementary Figure, https://stacks.cdc.gov/view/cdc/95822). Among 12 events (events 2–13) that occurred during students’ probable exposure periods, seven (events 3, 5, and 9–13) occurred 2–5 days before symptom onset or positive SARS-CoV-2 test results. Members of both teams mostly attended different events; however, three events (event 3 [a birthday party], event 5 [a dormitory or apartment visit], and event 11 [a coed soccer match]) most likely contributed to transmission because they were attended by members of both teams and persons not on the teams. The birthday party was attended by seven men’s team members (M1–M7), who reported wearing masks or social distancing <10% of the time and who all later received positive SARS-CoV-2 test results. Teammates were aware of two student athletes who were not on the soccer teams and who also received COVID-19 diagnoses after the event; investigators confirmed one positive SARS-CoV-2 test result using I-NEDSS. A dormitory or apartment visit (event 5) was attended by students M2–M7 and one women’s team player (W6), who later had a positive SARS-CoV-2 test result. Seven days later, these same students attended the coed soccer match (event 11) along with 21 teammates; five additional students later received a positive SARS-CoV-2 test result. Several other events occurred before the coed soccer match, including four lake gatherings (events 2, and 8–10), which also overlapped with students’ exposure periods and elicitation windows.

Twelve specimens collected from 10 students during days 23–33^§§^ were sequenced and found to be genetically similar, consistent with a single source of SARS-CoV-2 introduction, although the exact chain of transmission could not be ascertained. The sequences in this group belong to the same clade^¶¶^ known to be circulating in the Chicago area since March and related to viral sequences from New York.***

## Public Health Response

As part of university A’s response plan, symptomatic students were removed from play, received RT-PCR testing, and instructed to start isolation. Students with known or suspected exposure were quarantined separately and tested by RT-PCR. All soccer players living in on-campus dormitories were moved into quarantine dormitories to limit transmission between roommates. After completing isolation or quarantine periods, students could resume training sessions. As an additional mitigation strategy, the university implemented mandatory weekly viral SARS-CoV-2 testing with saliva specimens for all athletes, students living in campus housing, and those in the performing arts.

## Discussion

Several reports have described the challenges associated with SARS-CoV-2 transmission among college students who live and socialize together and have ongoing exposure on and off campus (*1*–*3*). This investigation identified 17 COVID-19 cases among students on a university’s men’s and women’s soccer teams who lived, trained, and socialized together. After commencement of training, numerous social events occurred. Little to no mask use or social distancing was reported at social events attended by symptomatic and asymptomatic students, which might have led to additional cases. Given the number of events during the investigation period, the precise event where transmission occurred cannot be determined and might have also occurred at an unreported event. Living in shared accommodations with persons who also participated in multiple social gatherings without complying with recommended prevention behaviors such as using masks might have compounded transmission risk within this group.

This outbreak highlights challenges to implementation of prevention strategies associated with persuading students at colleges and universities to adopt and adhere to recommended mitigation measures outside campus (*4*). University protocols mandated mask use during training sessions, and coaching staff members reported universal compliance. However, multiple students reported inconsistent mask use and social distancing at social gatherings, which quickly negated the benefits of pretraining testing, on-campus mask use, and social distancing prevention measures. Mask use was reported <10% of the time at the birthday party (event 3) and dormitory or apartment visit (event 5), and only one half of the students reported using masks >90% of the time during the coed soccer match (event 11). Consistent and correct mask use during gatherings can decrease transmission.^†††^ Encouraging students to wear masks and practice social distancing outside of official school activities might help prevent SARS-CoV-2 transmission among college students.^§§§^

A complementary prevention measure to mask use and social distancing could include periodic SARS-CoV-2 screening to identify presymptomatic, asymptomatic, or mildly symptomatic persons. For example, periodic testing of team members might have prevented nine players (M2–M7, W4, and W6–W7) (Supplementary Figure, https://stacks.cdc.gov/view/cdc/95822) from attending four social gatherings, had they been alerted of their test results and instructed to isolate.

The findings in this report are subject to at least four limitations. First, some students declined to provide contact information for family members and other close contacts apart from their teammates, limiting ability to assess the extent of secondary transmission. Second, although students were encouraged to refer to calendars, text messages, and social media to recall contacts and dates, many students reported difficulty remembering dates of symptom onset or events and size of gatherings. Third, not all specimens with a positive SARS-CoV-2 test result could be sequenced. Specimens not sequenced might be genetically dissimilar, which would suggest multiple sources of introduction within this group. Finally, estimated exposure periods and elicitation windows for asymptomatic persons might be inaccurate. Some students might have been infectious for >2 days before receiving a positive SARS-CoV-2 test result, limiting the ability to accurately identify all potential transmission events.

SARS-CoV-2 can quickly spread among college athletes. To control COVID-19 outbreaks on college campuses, more effective messaging and prevention strategies are needed to promote mask use and physical distancing in social settings. Also, findings support CDC considerations for institutes of higher education^¶¶¶^ regarding the utility of periodic repeat testing of persons with known or suspected exposure to COVID-19, persons with possible exposure in the context of an outbreak, and asymptomatic persons without known exposure. These strategies can help improve the timeliness of outbreak detection and inform control measures in settings with moderate to substantial community transmission.

**Summary**

**What is already known about this topic?**

SARS-CoV-2 transmission occurs in congregate settings, including colleges and universities.

**What is added by this report?**

Investigation of 17 COVID-19 cases among a university’s men’s and women’s soccer team identified numerous social gatherings as possible transmission events. Minimal mask use and social distancing resulted in rapid spread among students who live, practice, and socialize together.

**What are the implications for public health practice?**

Colleges and universities are at risk for COVID-19 outbreaks because of shared housing and social gatherings where recommended prevention guidance is not followed. Schools should consider conducting periodic repeat testing of asymptomatic students to identify outbreaks early and implementing policies and improving messaging to promote mask use and social distancing.

## Footnotes

* University policy limited on-campus congregating and gatherings and required mask use in all settings (except personal residence), social distancing, and daily health screening.

† The term “quarantine” is used to describe the 14-day period after a person who might have been exposed to COVID-19 stays away from others. The term “isolation” is used to describe the 10-day period when a person stays away from others after that person experiences symptom onset or receives a positive SARS-CoV-2 test result.

§ https://www.chicago.gov/content/dam/city/depts/cdph/HealthProtectionandResponse/CDPH%20Order%202020-2%20hospital%20duties%20SECOND%20AMENDED%20AND%20RE-ISSUED%20FINAL.pdf.

¶ Team members who did not participate in the voluntary training sessions and were not living in shared housing with other teammates during the investigation period were excluded from this investigation.

** 45 C.F.R. part 46.102(l)(2), 21 C.F.R. part 56; 42 U.S.C. Sect. 241(d); 5 U.S.C. Sect. 552a; 44 U.S.C. Sect. 3501 et seq.

†† A student’s elicitation window is the 2-day period when the student was infectious, before symptom onset (for symptomatic students with COVID-19) or a positive SARS-CoV-2 RT-PCR test result (for asymptomatic students with COVID-19), and not under isolation. For asymptomatic persons, the elicitation window is estimated. Persons are presumed to be infectious at the beginning of the elicitation window and are presumed not to be infectious at discontinuation of isolation. https://www.cdc.gov/coronavirus/2019-ncov/php/contact-tracing/contact-tracing-plan/investigating-covid-19-case.html.

§§ Two students with COVID-19 on the women’s team had two specimens available for sequencing. Fifteen specimens were available for sequencing; however, three specimens were removed because of low amount of sequence data that aligned to the reference genome. Specimens were not available for seven students with COVID-19.

¶¶ All filtered isolates were assigned to clade 20B on Nextclade. https://nextstrain.org/blog/2020-06-02-SARSCoV2-clade-naming.

*** https://www.medrxiv.org/content/10.1101/2020.05.19.20107144v2.

††† https://www.cdc.gov/coronavirus/2019-ncov/prevent-getting-sick/cloth-face-cover-guidance.html.

§§§ https://www.cdc.gov/coronavirus/2019-ncov/community/colleges-universities/considerations.html.

¶¶¶ https://www.cdc.gov/coronavirus/2019-ncov/community/colleges-universities/ihe-testing.html.
